# Family Level Phylogenies Reveal Relationships of Plant Viruses within the Order Bunyavirales

**DOI:** 10.3390/v12091010

**Published:** 2020-09-10

**Authors:** Venura Herath, Gustavo Romay, Cesar D. Urrutia, Jeanmarie Verchot

**Affiliations:** 1 Department of Plant Pathology & Microbiology, Texas A&M University, College Station, TX 77802, USA; venura.herath@tamu.edu (V.H.); gustavo.romay@tamu.edu (G.R.); curru001@tamu.edu (C.D.U.); 2 Department of Agriculture Biology, Faculty of Agriculture, University of Peradeniya, Peradeniya 20400, Sri Lanka

**Keywords:** *Bunyavirale*, RNA virus, emerging virus, virus evolution, plant virus, cophylogeny, hallmark genes

## Abstract

*Bunyavirales* are negative-sense segmented RNA viruses infecting arthropods, protozoans, plants, and animals. This study examines the phylogenetic relationships of plant viruses within this order, many of which are recently classified species. Comprehensive phylogenetic analyses of the viral RNA dependent RNA polymerase (RdRp), precursor glycoprotein (preGP), the nucleocapsid (N) proteins point toward common progenitor viruses. The RdRp of *Fimoviridae* and *Tospoviridae* show a close evolutional relationship while the preGP of *Fimoviridae* and *Phenuiviridae* show a closed relationship. The N proteins of *Fimoviridae* were closer to the *Phasmaviridae*, the *Tospoviridae* were close to some *Phenuiviridae* members and the *Peribunyaviridae*. The plant viral movement proteins of species within the *Tospoviridae* and *Phenuiviridae* were more closely related to each other than to members of the *Fimoviridae*. Interestingly, distal ends of 3′ and 5′ untranslated regions of species within the *Fimoviridae* shared similarity to arthropod and vertebrate infecting members of the *Cruliviridae* and *Peribunyaviridae* compared to other plant virus families. Co-phylogeny analysis of the plant infecting viruses indicates that duplication and host switching were more common than co-divergence with a host species.

## 1. Introduction

Viruses in the order *Bunyavirales* infect arthropods, plants, protozoans, and vertebrates. Their RNA genomes are segmented and exhibit negative or ambisense polarity. Each virus species has a fixed number of genome segments which range from two to eight, with plant viruses having the largest numbers of segments. The nucleotide sequences at the 3′ and 5′ terminus of each genome segment are complementary and form panhandle structures for stability. Their RNA segments are mostly coated in nucleocapsid proteins and further encapsulated in an envelope derived from its host cell.

*Bunyavirales* is a recently established taxonomic order that encompasses twelve families comprising 46 genera [1,2]. Four families contain members that cause life-threatening diseases in humans: *Hantaviridae*, *Nairoviridae*, *Peribunyaviridae* and *Phenuiviridae* [1,2]. These families include the species *Bunyamwera virus (BUNV*), *Crimean-Congo haemorrhagic fever virus (CCHFV)*, *Hantaan virus (HTNV)*, *La Crosse virus (LACV)*, *Rift Valley fever virus (RVFV)*, *Severe fever with thrombocytopenia syndrome virus (SFTSV)*, and *Sin Nombre virus (SNV).* Three families within *Bunyavirales* contain members that infect plants as their primary host: *Fimoviridae*, *Phenuiviridae*, and *Tospoviridae*. Within these families, there is one genus of plant infecting viruses: *Emaravirus*, *Tenuivirus*, and *Orthotospovirus*, respectively. Across *Bunyavirales*, viruses can have three segments of negative-sense or ambisense RNA that are named according to their relative length. These segments are known as large (L), medium (M), and small (S) which encode the viral RNA dependent RNA polymerase (RdRp), a polyprotein precursor glycoprotein (preGP) that is co-translationally cleaved into two mature glycoproteins (Gn and Gc), and the nucleocapsid (N) protein, respectively. Among the plant viruses with more than three genome segments where “x” equals the total number of segments, each segment is numbered from RNA1 to RNAx and are assigned sequentially to each segment in order of decreasing nucleotide length. A novel genus that is tentatively named *Coguvirus* has a bi-partite genome, lacks an external envelope and, is proposed to the order *Bunyavirales* [3,4].

The origins and evolutionary history of plant viruses within *Bunyavirales* are unclear. While phylogenetic studies suggest common ancestral origins of vertebrate and arthropod infecting viruses, the ancestral lineages of plant viruses within this taxonomic order have not been well studied [5,6]. Extensive sampling of arthropods (crustaceans, centipedes, insects, and spiders) have revealed new species of negative-sense RNA viruses, and many appear to be ancestral to viruses that cause diseases in vertebrate hosts [5,7,8,9,10]. Koonin and Dolja (2014) coined the term “hallmark genes” referring to viral genes that encode the necessary apparatus of viral replication and encapsidation and provide important clues about the evolutionary origins of disease-causing viruses. Studies of hallmark genes provide insight into the shared and conserved domain modules that are used in classification schemes to understand common evolutionary histories [11]. While there are extensive reports on the evolutionary relationships among positive-strand RNA and double-strand RNA viruses built on the analyses of viral hallmark genes, less is known about the evolutionary connections among the hallmark genes of plant-infecting viruses with negative sense or ambisense genomes, especially within *Bunyavirales* [4,12,13,14].

Recent research in the field of virus metagenomics has expanded the list of new plant-infecting species within *Bunyavirales*, which has contributed to the recent reorganization of families within this taxonomic order [2]. This study examines the phylogenetic lineages and host associations of recently discovered plant-infecting viruses within *Bunyavirales* by examining the shared and conserved hallmark genes among arthropod, plant, protozoan, and animal-infecting counterparts. This study also includes analysis of plant viral movement proteins which represent important changes in virus evolution from deeply rooted ancestral viruses.

## 2. Materials and Methods

### 2.1. Phylogenetic Analysis of Bunyavirales

We retrieved RdRp, preGP, N, and MP sequences from the NCBI protein archive (Supplementary Table S1). We used the updated taxonomy of the order *Bunyavirales* by the International Committee on Taxonomy of Viruses (ICTV) [2] as a guide to retrieve sequences of each representative virus species. Retrieved sequences were aligned using MAFFT ver. 7 [15,16,17] using E-INS-i algorithm. Ambiguously aligned regions were removed using the trimming mode ML_Automated1 of TrimAl ver. 1.3 wrapper embedded in TBTools ver. 1.0 [18,19]. ProtTest ver. 3.4.2 was used to determine the best candidate of the amino acid substitution models for all sequence alignments. LG+I+G+F, LG+G+F, LG+G and LG+I+G+F amino acid replacement models were used for the phylogenetic analysis of RdRp, NC, GP and MP respectively [20]. Phylogenetic trees were generated using PhyML program ver. 3.1 with the maximum likelihood (ML) approach embedded in SeaView ver. 5.0.4 [21,22]. Tree searching was employed using the nearest neighbor interchange (NNI) search strategy. Branch support was computed using an approximate likelihood ratio test (aLRT) with the Shimodaira–Hasegawa-like (SH) procedure. Phylogenetic trees were visualized using iTOL server ver 5.6 [23,24]. Images were compiled using Adobe Photoshop CC (ver. 21.2.0).

### 2.2. Analysis of the Untranslated Regions (UTRs) of RNA Segments

The 3′ and 5′ UTR regions of viral segments were extracted using NCBI nucleotide database (Supplementary Tables S2 and S3). Sequences were manually checked using RNAfold ver. 2.4.14 [25] plugin built into Geneious Prime ver. 2020.2 for sequence quality and completeness. Then the first 20 nucleotides were extracted using the same program. Sequence logos were created using the WebLogo 3 server [26,27]. Images were compiled using Adobe Photoshop CC (ver. 21.2.0).

### 2.3. Pairwise Sequence Alignment and Identity Score Calculation

For calculating identity scores of MP amino acid sequences, pairwise sequence alignment was performed using the software Sequence Demarcation Tool (SDT) v. 1.2 [28].

### 2.4. Co-Phylogenetic Analysis

Cophylogenetic relationships between families and their natural hosts were analyzed with event-based co-phylogeny analysis tool Jane ver. 4.01 [29]. Phylogenetic relationships among the hosts were obtained from the NCBI Taxonomy browser [30]. The host information was obtained from the Virus–Host DB [31] and available literature [32] (Supplemental Table S4). Phylogenies of virus families were conducted based on the RdRp protein sequences as described above. Viruses without host information were excluded from the analysis. The following cost scheme was used for the analysis in Jane; co-divergence = 0, duplication = 1, host switch = 1, loss = 1, failure to diverge = 1. The number of generations and the population size was both set to 100. In order to visualize the taxonomic relationships between plant and insect taxa, we used concatenated genomic segments (L, M, S, and RNA 4 segments) containing four hallmark genes (RdRp, NC, GP, and MP) of plant viruses. Viruses with missing segments and incomplete sequences were excluded from the analysis. The sequence concatenation was carried out using Geneious Prime version 2020.2.1. Concatenated sequences were aligned using MAFFT server version 7 [17] using E-INS-i method [16]. A neighborhood joining tree was generated using the conserved sites (1800 nts) using Jukes–Cantor substitution model with 1000 bootstraps using MAFFT server version 7 [16]. Plant host taxonomies were obtained from APWeb version 14 [33,34]. The resulting phylogenetic tree was visualized and color-coded in iTOL server version 5.6 [23,24]. Image compilation was carried out in Photoshop CC version 21.2.0 and Illustrator version 24.2.3.

## 3. Results

### 3.1. Phylogeny and Domain Analysis of RNA-Dependent RNA Polymerase (RdRp), Glycoprotein Precursor (preGP), Nucleocapsid Proteins (N), and Movement Proteins (MP) of Bunyavirales Members

#### 3.1.1. Phylogeny of RdRp

**Figure 1:**
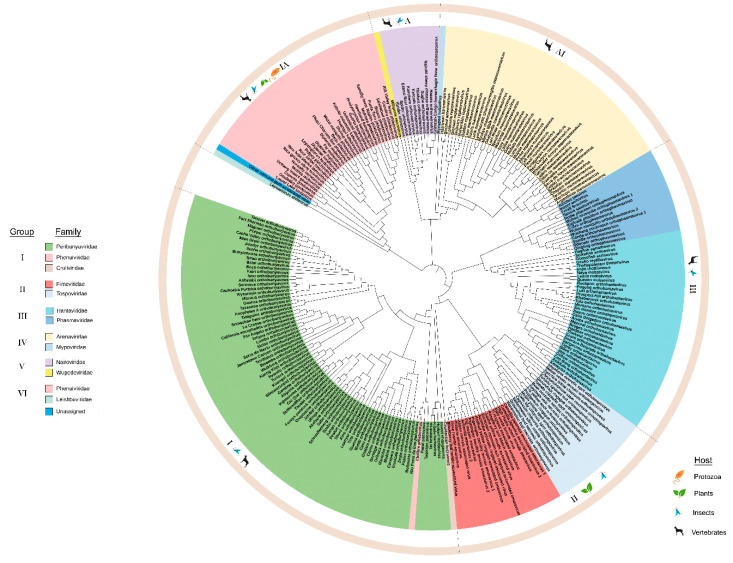
Maximum likelihood phylogenetic tree of the amino acid sequences of the RNA-dependent RNA polymerase (RdRp). The virus families are color-coded and the hosts for viruses within each group are indicated in the outermost circle. The six groups are identified in the legend and the boundaries of these groups are indicated in the outer ring of the phylogeny. Group I: *Peribunyaviridae*, *Phenuiviridae*, and *Cruliviridae*. Group II: *Fimoviridae* and *Tospoviridae*. Group III: *Hantaviridae* and *Phasmaviridae*. Group IV: *Arenaviridae* and *Mypoviridae*. Group V: *Nairoviridae* and *Wupedeviridae*. Group VI: *Phenuiviridae*, *Leishbuviridae*, and unassigned species. Clade validation is based on the approximate likelihood ratio test (aLRT)-Shimodaira–Hasegawa (SH)-like test values.

For all negative-strand RNA viruses in the order *Bunyavirales*, RNA1, (or the L segment) is the longest and encodes RdRp. The RdRp sequences for 253 species belonging to arthropod, plant, protozoan, and vertebrate infecting viruses within *Bunyavirales* were compiled (Supplementary Table S1) to build an ML phylogeny. The ML tree in Figure 1 covers 12 families and one unassigned species and, has three deeply rooted clades with viruses of insect hosts at the basal position as reported in Guterres et al. (2017) [6]. Within these three clades are six major lineages that we identified as groups I through VI (Figure 1). These groups are recognized based on the cluster of branches emanating from the most distant node, suggesting a common lineage progenitor. These lineage groups are supported by their primary hosts (protozoa, plant, arthropod, and vertebrate). Except for group II, all other groups contain families that infect vertebrates and/or invertebrates. Notably, the species *Chilibre phlebovirus* (CHIV) is classified by the ICTV as a member of the family *Phenuiviridae* but the ML tree indicates that the RdRp is in the lineage group I with *Peribunyaviridae* family and clusters with the *Pacuvirus* and *Herbevirus* genera. This unusual relationship, verified using the aLRT-SH test (Supplementary Figure S1), suggests that the taxonomic assignment of CHIV may be erroneous.

Viruses of lineage groups I, II, and III traces to one deeply rooted clade (Figure 1) identified by Guterres et al. (2017) as a Bunyavirus-like supergroup [6]. The deepest root of this clade leads to group III viruses that include the *Orthophasmavirus*, *Jonvirus*, and *Feravirus* genera. The *Orthohantavirus* genus is the next bifurcation in Group III. Within this large clade is another deep root that bifurcates to group II plant-infecting *Orthotospovirus* and *Emaravirus* and the larger group I *Orthobunyavirus* and *Lincruvirus* genera. The species *Crustacean lincruvirus* is at the root of the group I *Orthobunyavirus* lineage [9]. Emaraviruses are vectored by mites and orthotospoviruses are vectored by thrips [12,35,36].

The next deeply rooted clade includes groups IV and V, *Arenavirus* and *Nairovirus*. This is known as the arenanairo-like virus superclade according to Guterres et al. (2017) [6]. The invertebrate-infecting species *Myriapod hubavirus*, *Haartman hartmanvirus*, and *Striated antennavirus* are at the deepest root supporting lineage group IV viruses. The invertebrate-infecting species *Millipede wumivirus* is at the deepest root supporting lineage group V.

The third major branch has the invertebrate-infecting species *Leptomonas shilevirus* and *Laurel Lake virus* at the deepest node. The plant-infecting genera *Tenuivirus* and *Coguvirus* and the insect-infecting genus *Goukuvirus* are the closest relatives to these invertebrate-infecting genera [3]. While Guterres et al. (2017) identified this as a phlebo-like virus superclade, the *Phlebovirus* genus represents a smaller fraction of viruses within this lineage group with the majority of viruses representing plant, insect, and protist-infecting viruses [6]. The RdRps of the plant-infecting virus species within this phylogeny, like the vertebrate-infecting viruses, appear to have arisen from common progenitor viruses [37].

**Figure 2:**
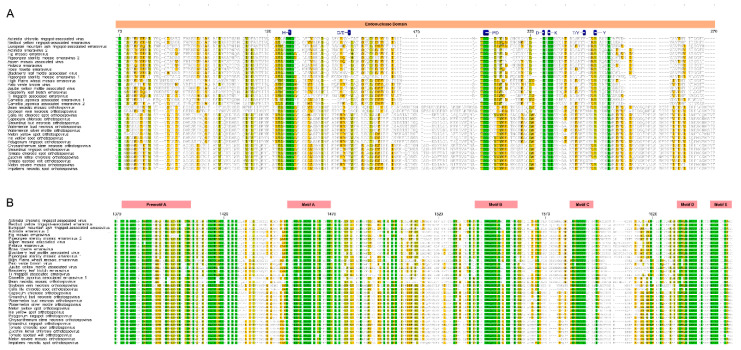
Amino acid alignment showing conserved motifs of the RdRp within *Fimoviridae* and Tospoviridae. (**A**). The endonuclease domain is indicated by pink bar and active site motifs are identified in blue. (**B**). The polymerase function motifs are named in the red bars as preA motif through E motif. The alignment colored based on the sequence similarity.

Considering the close relationship between the families *Fimoviridae* and *Tospoviridae*, we carefully examined an alignment of their RdRp sequences. Crystal structures of several members of the order *Bunyavirales* have been used to identify functional motifs and similarities across species within the order, and these reports informed this analysis [38,39,40]. The N-terminal domain harbors the endonuclease activity required for cap-snatching processes (Figure 2A). The polymerase domain near the C-terminus has motifs preA and A through E which are conserved in linear arrangement and distance (Figure 2B). Members of the *Fimoviridae* and *Tospoviridae* share the conserved motifs (H…PD…D/E…K… T/Y…Y) in the endonuclease active center occurring in all families of *Bunyavirales* [35,38,40], but with a few modifications. First, species within the *Orthobunyavirus*, *Orthotospovirus*, *Hantavirus*, and *Phlebovirus* genera have the conserved D/E motif between the H and PD (Figure 2A, position 145 in the alignment) that also occurs in members of the genus *Orthotospovirus* but is missing in members of the genus *Emaravirus* [35,38,40]. The T/Y at position 225 is reported as T/K for members of the *Orthobunyavirus*, *Hantavirus*, and *Phlebovirus*. This alignment shows the T/K is conserved at position 225 for *Fimoviridae* and *Tospoviridae* members. Orthotospoviruses have two added sequences between positions 165 and190, and between 242 and 254 (Figure 2). The C-terminal polymerase domain is highly conserved between *Fimoviridae* and *Tospoviridae*. The motifs preA, A through E have a high proportion of identical and highly conserved residue with only a few minor changes that differentiate members of the genera *Emaravirus* and *Orthotospovirus*. One minor difference occurs in the preA motif at position 1388 to 1390; *Fimoviridae* has a tripeptide that is NxQ while *Tospoviridae* has SMK. In motif A, at position 1452 to 1455, *Fimoviridae* has LSSD and *Tospoviridae has* LSAD. At position 1500 to 1510, which is between motifs A and B, emaraviruses have IxLTDxxN/DxF and orthotospoviruses have VCIPTDIFLNL. Then, at position 1581 in motif C, emaraviruses have S/F/Y while orthotospoviruses have W.

#### 3.1.2. Phylogeny of preGP

The *Bunyavirales* RNA2 (or M segment) encodes the preGP which is inserted into the host endoplasmic reticulum (ER) and cleaved by the cellular signalase into Gn and Gc [41]. The mature Gn and Gc are required for virus particle budding and entry into target cells. Most virus members also encode one or more nonstructural proteins including the major nonstructural protein (NSm) which are positioned in one of five locations within the RNA2 or M segment (Supplementary Figure S2). In general, the NSm of vertebrate-infecting viruses is required for virus growth in cell cultures but is dispensable for virus replication. For plant-infecting viruses, NSm is required for virus cell-to-cell spread. For *Nairoviridae* and *Peribunyaviridae*, the NSm is part of the polyprotein located between the Gn and Gc regions. For *Phasmaviridae*, NSm is located at the N-terminus of the Gn sequence. For *Tospoviridae*, the NSm is ambisense oriented and located next to the Gc domain. For *Phenuiviridae*, the NSm is an open reading frame nested within the Gn region. Members of the genera *Tenuivirus* and *Emaravirus* lack the NSm coding sequence in RNA2 and M segment (Supplementary Figure S1). The tenuiviruses and emaraviruses have more than three genome segments, and their MP is located on another genome segment.

**Figure 3:**
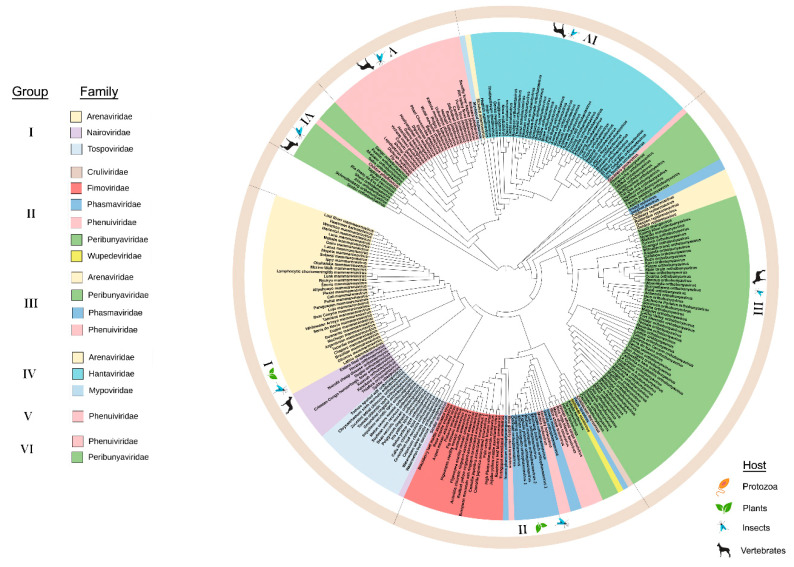
Maximum likelihood phylogenetic tree of the amino acid sequences of the glycoprotein precursor (preGP). Six groups were identified based on clustering from the most distant node. The legend identifies the lineage groups and colors used to identify taxonomic families as in Figure 1. Group I: *Arenaviridae*, *Nairoviridae* and *Tospoviridae*. Group II: *Cruliviridae*, *Fimoviridae*, *Phasmaviridae*, *Phenuiviridae*, *Peribunyaviridae*, and *Wupedeviridae.* Group III: *Arenaviridae*, *Peribunyaviridae*, *Phasmaviridae*, and *Phenulviridae*. Group IV: *Arenaviridae*, *Hantaviridae*, and *Mypoviridae*. Group V: *Phenuiviridae*. Group VI: *Phenuiviridae* and *Peribunyaviridae*. Families are color-coded and the hosts for viruses within each group are indicated in the outermost circle. Clade validation is based on the aLRT-SH-like test values.

The preGP phylogeny shows three deeply rooted branches and displays six major lineage groups comprising 11 taxonomic families (Figure 3). These lineage groups are supported by their primary host (vertebrate plant, and insect). The *Leishbunyaviridae,* and unassigned *Coguvirus* were not included because the full-length sequences for the M segment (RNA 2) are not available. The *Gouleako goukuvirus,* which is the type member of the genus *Goukuvirus* (family *Phenuiviridae*), is a deeply rooted branch that precedes the major subclades in groups I, II, and III. Looking at the M segment (or RNA2 segment) for each genus within these virus families, the length of the preGP varies significantly. In group I, *Orthonairovirus* fall into two classes that either contain or lack the NSm within the polyprotein (Supplementary Figure S2). The members of the plant-infecting virus genus *Orthotospovirus* encode NSm in an ambisense direction which does not overlap the glycoprotein precursor. In group II, only members of the genus *Feravirus* contain an NSm sequence, however, this does not overlap the glycoprotein precursor. The plant-infecting members of *Emaravirus* and *Tenuivirus* do not encode NSm (Supplementary Figure S2). Among group III, the NSm adjacent to the Gn domain of the polyprotein for *Orthobunyavirus*, *Shangavirus*, and *Jonvirus*. It is reasonable to suggest that the NSm likely influenced the diversification of some viral preGPs within the ML tree, but given the diversity of the preGPs, there are likely to be other factors affecting their evolution (Supplementary Figure S2).

Two deeply rooted branches lead to the group VI and group V, the primarily arthropod-borne species of *Peribunyaviridae* (*Orthobunyavirus* and *Pacuvirus*) and *Phenuiviridae.* From the *Pacuvirus* branch, there are three major subclades: three species of *Orthobunyavirus* cluster in group VI, the group V *Phenuiviridae* cluster, and the group IV cluster of primarily *Hantaviridae* with sole representatives of *Arenaviridae* and *Mypoviridae* (Figure 3). Among group V, some phleboviruses such as *Rift valley fever virus*, contain the NSm as a nested gene overlapping the Gn domain of the polyprotein coding sequence (Supplementary Figure S2). NSm has not been identified among Group IV and V viruses.

#### 3.1.3. Phylogeny of Nucleocapsid (N) Proteins

**Figure 4:**
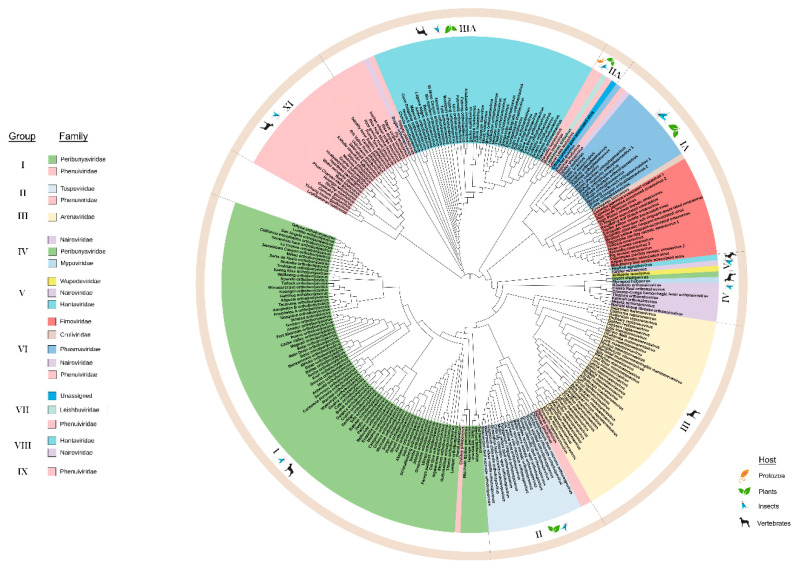
Maximum likelihood phylogenetic tree of the amino acid sequences of the nucleocapsid (N). Ten lineage groups were identified based on clustering from the most distant node. Group I: *Peribunyavirdae* and *Phenuiviridae*. Group II: *Tospoviridae* and *Phenuiviridae*. Group III: *Arenaviridae*. Group IV: *Nairoviridae*, *Peribunyaviridae*, and *Mypoviridae*. Group V: *Wupedeviridae*, *Nairoviridae*, and *Hantaviridae*. Group VI: *Fimoviridae*, *Cruliviridae*, *Phasmaviridae*, *Nairoviridae*, and *Phenuiviridae*. Group VII: *Leishbuviridae*, *Phenuiviridae*, and unassigned species. Group VIII: *Hantaviridae* and *Nairoviridae*. Group IX: *Phenuiviridae*. Group X: *Phenuiviridae*. Families are color-coded and the hosts for viruses within each group are indicated in the outermost circle. Clade validation is based on the aLRT-SH-like test values.

The N proteins of 268 species within the order *Bunyavirales* were used to construct an ML tree with three deeply rooted branches. We identified ten lineage groups (Figure 4) and seven of these groups comprise two or more taxonomic families. Group III contains only *Arenaviridae* and groups IX and X contain only *Phenuiviridae*. Group IX and X include vertebrate and insect-infecting members of *Phenuiviridae*. One similarity between the N and RdRp phylogenies is that the CHIV clusters with the *Pacuvirus* and *Herbevirus* genera in lineage group I along with the family *Peribunyaviridae* (Supplementary Figure S1). Locating CHIV in group I suggests that its ICTV taxonomic classification may be erroneous [42]. 

One deeply rooted branch leads to lineage group VII and subsequent subclades arising from this branch include lineage groups I through VI. This large clade spanning from groups I to VII includes the families *Peribunyaviridae*, *Phasmaviridae*, *Fimoviridae*, *Phenuiviridae*, *Tospoviridae*, *Arenaviridae*, and *Nairoviridae* (Figure 4). This deep branch leads directly to Group VII viruses which include the unassigned *Coguvirus*, *Leishbuviridae*, and *Phenuiviridae* members that infect protozoa, arthropods and plants. Each subclade includes an arthropod-infecting genus except for group III Arenaviridae which are vertebrate infecting viruses. For example, the *Herbevirus* genus of group I viruses infects mosquitoes. There are two insect-infecting members of the *Phenuiviridae* in group II that likely gave rise to *Tospoviridae*. Groups IV, V, VI, and VII have the deepest branches associated with protozoan or arthropod infecting viruses. A large component of group VIII includes *Hantaviridae*, insect and plant-infecting *Phenuiviridae*, and one *Nairoviridae* member. 

### 3.2. Phylogeny of MP of Plant Virus Genera Orthotospovirus, Emaravirus and Tenuivirus

**Figure 5:**
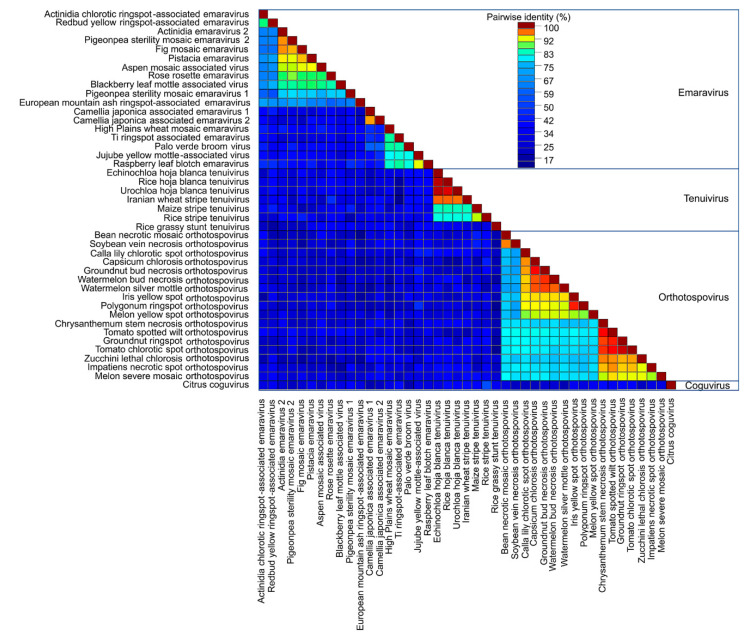
Pairwise sequence alignment of movement proteins (MP) for plant viruses within *Bunyavirales*. Sequence analysis was conducted for all available plant virus within *Bunyavirales*. The plant virus families are *Emaravirus*, *Tenuivirus*, *Orthotospovirus*, and *Coguvirus*. The alignment is colored based on the sequence similarity.

Plant virus genomes encode MPs that facilitate intercellular movement and long-distance movement through the vasculature. Researchers identified the *Emaravirus* RNA4 that encodes the 42 kDa P4 protein [43,44], the *Tenuivirus* NS4 [12,45,46], and the *Orthotospovirus* NSm protein as the viral MPs. Previous sequence and structural analysis determined these proteins affiliate with the ‘30K superfamily’ of viral MPs which contain a conserved core of mostly beta-strands [47]. Pairwise comparisons of 42 MP sequences showed most species within the *Emaravirus*, *Tenuivirus*, or *Orthotospovirus* genera shared 60–100% identity and had fewer common residues between the genera (Figure 5). *Emaravirus* MPs formed three subgroups (Figure 4). The first subgroup shares more than 75% identity and includes the species *Ti ringspot associated virus*, *Palo verde broom virus*, *Jujube yellow mottle associated virus*, and *Raspberry leaf blotch virus*. The second subgroup includes *Camellia japonica associated viruses 1* and *2*, and *High Plains wheat mosaic virus*. The third subgroup includes 11 species that share 60% or more identity: *Actinidia chlorotic ringspot-associated virus*, *Redbud yellow ringspot-associated virus*, *Actinida virus 2*, *Pigeonpea sterility mosaic virus 1* and *2*, *Fig mosaic virus*, *Pistacia virus*, *Aspen mosaic associated virus*, *Rose rosette virus*, *Blackberry leaf mottle-associated virus*, and *European mountain ash ringspot-associated virus*. Among tenuiviruses, the *Rice grassy stunt virus* shared less than 50% identity with other genus members. There were two groups of orthobunyaviruses that shared more than 80% identical residues (Figure 5).

**Figure 6:**
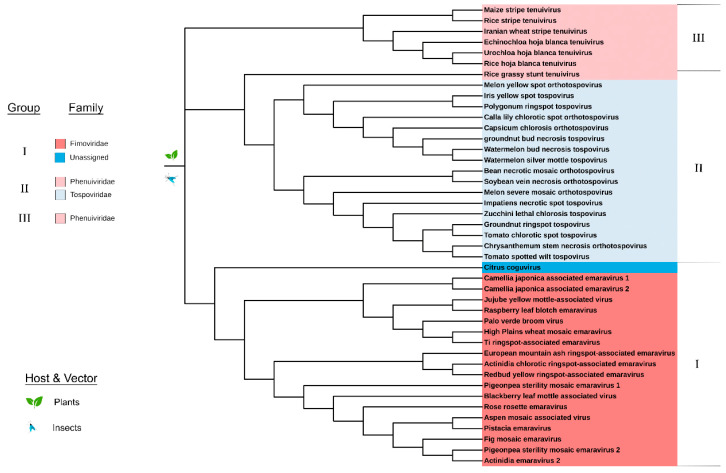
Maximum likelihood phylogenetic tree of the amino acid sequences of the movement protein (MP) belonging to plant viruses. Three groups were identified based on clustering from the most distant node: Group I: *Fimoviridae* and an unassigned species. Group II: *Phenuiviridae* and *Tospoviridae*. Group III: *Phenuiviridae*. All viruses of *Bunyavirales* with an available MP sequence have plant hosts. Clade validation is based on the aLRT-SH-like test values.

An ML tree showed the MPs in three major clades. Group I consists of the *Fimoviridae* and *Coguvirus* MPs. Group II contains the *Tospoviridae* as well as the *Rice grassy stunt tenuivirus* MPs. Group III is comprised of MPs belonging to *Phenuiviridae* (Figure 6). Conserved structural features of viral movement proteins within the 30K superfamily have been well studied [44,47,48]. Given the number of newly identified species of plant-infecting viruses of *Fimoviridae*, *Phenuiviridae*, and *Tospoviridae*, the multiple sequence alignment shows a low percentage of conserved residues (~18%) across all families (Supplementary Figure S3). Since there is a prevalence of hydrophobic residues (Φ) across the sequences, we manually reviewed the alignment to look for obvious patterns. Notably, all 30K superfamily members have a conserved aspartic acid (D) residue that is found in these 42 movement proteins and is referred to as the “D motif” [47,48]. We determined that the emaraviruses and orthotospoviruses have a common motif surrounding the D motif: Φ-X-Φ-P-X_(14)_-D-X_(52–63)_-W, while the tenuiviruses have a submotif Φ-X-Φ-P-D. The W residue is not conserved downstream of the D motif in the tenuivirus MPs (Supplementary Figure S3). 

### 3.3. Common Features of Complementary 3′ and 5′ Terminal Regions of Genome Segments

**Figure 7:**
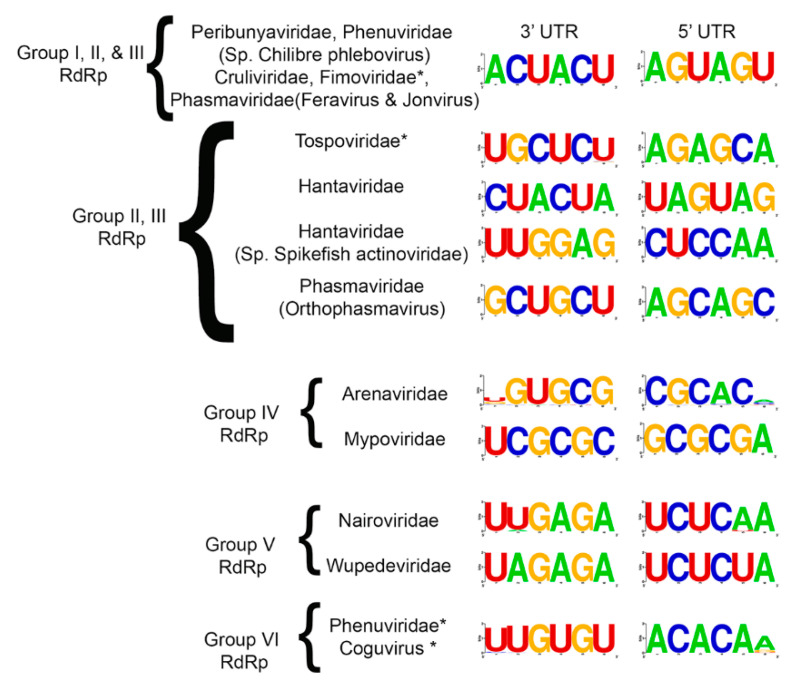
Consensus nucleotide sequence of the 3′ and 5′ termini for each genomic segment of *Bunyaviriales.* The consensus sequences were generated using the 6 most distal nucleotides on each end of the viral genomic segments. Each of the analyzed regions was located within a UTR. Families that contain plant viruses are highlighted with an asterisk.

The coding regions of each genome segment lie between terminal non-translated sequences that vary in length. The 3′ and 5′ genomic RNA termini are essential for RNA synthesis and are typically invariant. We compiled the terminal 20 nucleotides for all species that were used in the phylogeny into a table, leaving gaps for those whose sequences were not reported (Supplementary Table S2). We then trimmed the sequences to the first six nucleotides (Supplementary Table S3) and determined these are largely identical within each genus. Sequence logos were created for each family and there was a remarkable level of sequence identity within virus families (Figure 7). The most striking observation was that the 3′ and 5′ UTRs for *Peribunyaviridae*, *Cruliviridae*, the plant-infecting *Fimoviridae*, and two genera of *Phasmaviridae* (*Feravirus* and *Jonvirus*) had identical terminal sequences. It is interesting to see such conservation among animal, plant, and arthropod-infecting viruses. The species CHIV within the *Phenuiviridae*, which we repeatedly noted to be misclassified phylogenetically with *Peribunyaviridae*, also shares the identical terminal sequences with these virus families. Additionally, the plant-infecting *Phenuiviridae* (*Tenuivirus* genus) and *Coguvirus* share identical 5′ ACACAA/G and 3′ U/AUGUGU terminal sequences.

The terminal nucleotides for *Tospoviridae*, *Arenaviridae*, and *Myopviridae* are unique (Figure 7). Notably, the *Orthophasmavirus* differs from *Feravirus* and *Jonvirus* in that they each have mirrored tri-nucleotide repeats but differ by a single conserved nucleotide in each repeat. Where *Feravirus* and *Jonvirus* have 5′ AGUAGU and 3′ ACUACU, *Orthophasmavirus* has 5′ AGCAGC and 3′ GCUGCU (the unlike nucleotides are underlined). It is also worth noting that there is only one nucleotide difference between the 5′ and 3′ terminal sequences of *Nairoviridae* and *Wupedeviridae*. The *Nairoviridae* has 5′ UCUCAA and 3′ UUGAGA while *Wupedeviridae* has UCUCUA and UAGAGA. 

### 3.4. Cophylogenetic Analysis and Host Range Evolution

**Figure 8:**
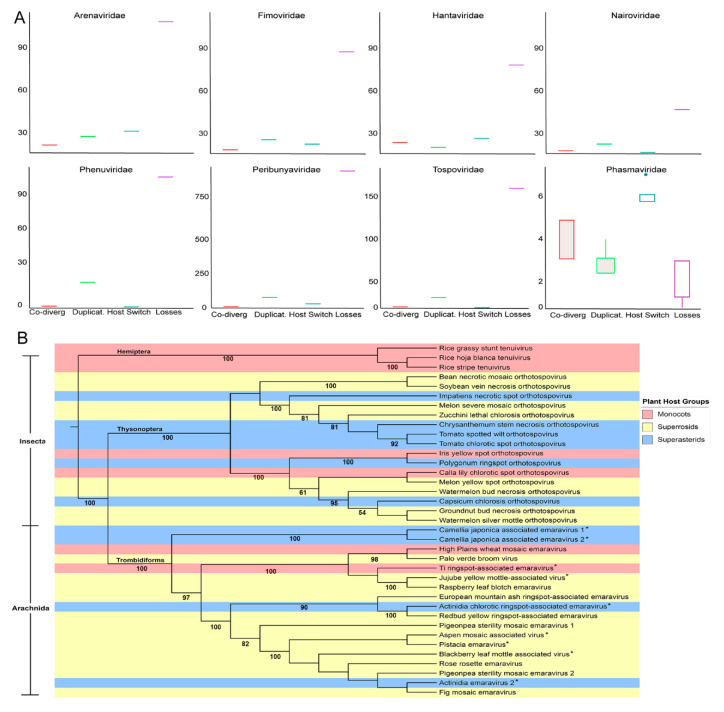
Estimation of co-phylogenetic events of the nucleic acid sequence of plant-infecting virus families within *Bunyavirales*. (**A**). The cophylogeny relationship is based on the RdRp sequences and analyzed using Jane ver. 4.0.1. (**B**). A neighbor joining tree generated using concatenated genomic segments containing RdRp, NC, GP, and MP. Color was used to identify host plant taxonomies and insect vector taxonomy is provided along the branches. Bootstrap values are provided.

Considering the distribution of host taxa on each ML tree, we performed co-phylogeny analysis of virus and host phylogenies at the species level (Figure 8A). These data revealed that duplication and host switching, otherwise known as cross-species transmission, are more common among *Arenaviridae*, *Fimoviridae*, *Hantaviridae*, and *Phasmaviridae* than co-speciation (also known as co-divergence). Duplication is more common than co-speciation or host switching for *Arenaviridae*, *Fimoviridae*, *Nairoviridae*, *Peribunyaviridae*, *Phenuiviridae* and *Tospoviridae*. Considering the preGP, N protein, and MP phylogenies show that vertebrate and plant infecting viruses are related to arthropod infecting viruses suggesting that cross-species transmission may occur between arthropod species, plant species or vertebrate species. However, there is little evidence to suggest the cross-kingdom movement of viruses. The tree also revealed between plant and vertebrate hosts but clustering, host switching during evolutionary history could support the divergent phylogenetic positions for some species within the taxonomic families. Surprisingly, the analysis suggests extinction plays a major role in the evolutionary history for all families in *Bunyavirales* except for *Phasmaviridae (*Figure 8A). The high losses could indicate that there was a mismatch between the independent host and virus phylogenies or descendent of the host species did not inherit a susceptibility to this virus. 

To better understand the links between plant infecting viruses, their arthropod vectors, and their plant hosts, an ML tree was generated using concatenated RNA segments representing hallmark genes and MP comprising the genera *Tenuivirus*, *Orthotospovirus*, and *Emaravirus.* Looking at the host spectrum, these plant virus genera are relatively restricted (Figure 8B). Tenuiviruses infect monocot hosts and do not associate with other host types and are transmitted by hemipteran insects. The orthotospoviruses and emaraviruses generally infect members of two large clades of flowering plants known as superrosids and superastrids. Both superrosids and superastrids arose around the same period of rapid evolutionary diversification of eudicots [33,49]. There are two examples of orthotospovirus and emaravirus species infecting monocots. The orthotospoviruses are transmitted by thysonopteran insects and emaraviruses are vectored by trombidiform mites. These plant virus taxa exhibit relatively restricted host and vector spectrum despite the examples of host-switching and low levels of virus-host co-divergence. These data suggest a long-term association between these plant viruses and their hosts although cross-species transmission occurs with some frequency.

A. *Estimation of phylogenetic events within the RdRp*. The amino acid sequence of the RdRp for each family of plant-infecting virus within the order *Bunyavirales* was analyzed and an estimate of co-divergence events (red), duplication events (green), host switch events (blue) and loss events (purple) were summed for each family. Boxes represent the estimated median (center line) interquartile range (IQR) and whiskers represent 1.5× IQR.

B. Maximum-likelihood *tree of viral* segments harboring hallmark genes and movement protein.

A maximum-likelihood phylogenetic tree was constructed from the complete viral genome segments that encode hallmark genes and movement protein (if the sequence is available) for each plant-infecting virus within the order *Bunyavirales*. The genome segments were concatenated in silico before analysis. The virus’ vector is listed to the left of the tree, and species with an asterisk (*) have an unknown vector. Each virus is color-code based on its plant host type: monocots (red), superrosids (yellow), or superasterids (blue).

## 4. Discussion

This study examines the phylogenetic placement of plant viruses within the order *Bunyavirales*. We focused on the genome segments L (or RNA1), M (or RNA2), and S (or RNA3) encoding the RdRp, preGP, and N proteins respectively because they consistently define all members of *Bunyavirales*. We included the analysis of the viral MP because they are a defining feature of plant infecting viruses. This research is timely because, in 2019, the order *Bunyavirales* was amended with significant changes in the associated numbers of families, genera, and species [2]. The ML trees of RdRp, preGP, and N proteins commonly show three deeply rooted branches extending from the base.

The RdRp ML tree shows the plant-infecting *Fimoviridae* and *Tospoviridae* in group II share a common node with *Peribunyaviridae* in group I. While the relatedness of *Tospoviridae* and *Peribunyaviridae* RdRps have been previously reported [6,50], this phylogeny highlights the close relatedness of the *Fimoviridae* and *Tospoviridae* RdRps. The RdRp amino acid sequence alignment shows that the *Orthotospovirus* and *Emaravirus* RdRps share a remarkably high level of conserved residues within the endonuclease and polymerase motifs and that the linear distance between these motifs is similar. These data suggest that selection pressures constrained the amino acid substitutions within these motifs [40].

The RdRp and N form a highly stable complex with viral RNAs that are packaged into virions [40]. The initiation of virus replication requires the formation of a replicative complex that includes the viral RdRp and N proteins. The complementary 3′ and 5′ UTRs of the viral RNA are important for the initiation of replication. The N protein disrupts hydrogen bonding of the “panhandle” structure and enables RNA synthesis by the RdRp [40,51,52]. Given the important engagement between the RdRp, N and UTR regions of the viral RNAs, we expected the N proteins to have similar evolutionary constraints as the RdRp. We were surprised to observe that the N proteins are not as closely related between members of the *Fimoviridae* and *Tospoviridae.* The ML phylogeny of the N proteins showed that the *Tospoviridae* and *Peribunyaviridae* share a common node that bifurcates to groups I and II, while *Fimoviridae* and the arthropod infecting *Phasmaviridae* share a common node in group VI. The complementary 3′ and 5′ termini of the genomic RNA showed a clearer pattern of co-divergence with the lineage groups represented in the RdRp phylogenies. For example, the *Peribunyaviridae*, *Cruliviridae*, *Fimoviridae*, two genera of *Phasmaviridae* (*Feravirus* and *Jonvirus*) and the *Chilibre phlebovirus* share identical terminal 6 nucleotides and the RdRps reside in Groups I, II, and III which derive from a common deep-rooted branch. The *Nairoviridae* and *Wupedeviridae* in Group V RdRp have identical termini except for one nucleotide and the RdRp Group VI *Phenuiviridae* and *Coguvirus* share identical termini. The RdRp Group II and III affiliated *Tospoviridae, Hantaviridae*, and *Phasmaviridae* have unique terminal sequences that are shared within these taxonomic families. It is also interesting to point out that the 3′ and 5′ terminal sequences of the plant infecting *Fimoviridae* share identity with the vertebrate infecting *Peribunyaviridae* and not the plant infecting *Tospoviridae*. This observation suggests that the high degree of sequence identity within the RdRp endonuclease and polymerase catalytic motifs of the *Fimoviridae* and *Tospoviridae* is not the driving force for co-evolution of the terminal UTR sequences [53]. However the sequences within neighboring regions of the UTRs that are likely important for replication, transcription, and translation might be influenced by the affinity of the N protein or host factors [38]. For the plant infecting viruses of *Fimoviridae*, *Tospoviridae*, *Phenuiviridae*, and the unassigned *Coguvirus*, experiments are needed to understand how the functional roles of the terminal UTRs.

The CHIV is assigned to the genus *Phlebovirus*; family *Phenuiviridae*. Members of the genus *Phlebovirus* are viruses that are borne by ticks, mosquitoes, and sandflies. Prior molecular characterization suggested that CHIV may be more related to the genus *Pacuvirus* within *Peribunyaviridae* [54]. Our ML analysis using a much larger dataset indicates that CHIV RdRp and N proteins share a specific relationship with the *Pacuvirus* within the Group I *Peribunyaviridae.* The preGP also shows a strong phylogenetic relationship with *Pacuvirus* and *Orthobunyavirus* in group VI. This ML analysis supports the suggestion that the taxonomic identity for CHIV should be moved from the *Phlebovirus* to the *Pacuvirus* genus within *Peribunyaviridae* [54]. Inter-lineage reassortment, although unlikely, may only be considered for the assignment of *Chilibre virus* because these viruses share common reservoirs [55]. However, it is unknown whether an RdRp of one virus can support the replication of such distantly related viruses within this order, arguing against heterotypic reassortment [56,57,58].

Interestingly, the preGP and N protein phylogenies each exhibited a higher extent of diversity with members of the same taxonomic family sometimes represented in more than one lineage group. For *Phenuiviridae*, the preGP resides in four groups while the N proteins reside in six different groups. Focusing on the plant infecting viruses, the *Tenuivirus* N proteins extend from a deep branch which at its base bifurcates to the insect and vertebrate infecting *Webuvirus*, *Pidchovirus*, and *Orthohantavirus* [59]. The phylogenetic positions of the *Emaravirus* and *Orthophasmavirus* preGP and N proteins suggest a similar ancestry. The *Orthotospovirus* preGP proteins are phylogenetically positioned near the *Orthonairoviruses* and *Mammarenaviruses* while the N proteins extend from a node that is affiliated with two dipteran infecting virus members of *Phenuiviridae* in group II [6]. These observations suggest that the evolutionary connections among viruses with *Bunyavirales* involve a network of gene exchanges. Such gene exchanges likely led to the emergence of new virus species. The data in Supplementary Figure S1 highlight the varying sense and ambisense positions of the preGP open reading frames associated with *Arenaviridae* and *Phenuiviridae* within several phylogenetic groups and strongly suggests the exchange of genes between viruses. An evolutionary mechanism of recombination is supported by the presence or absence of NSm either fused or nested within the preGP coding sequences of neighboring virus genera within a phylogenetic group.

Analysis of the plant viral MPs shows three lineage groups and surprisingly the MPs of *Rice grassy stunt tenuivirus* and orthotospoviruses are closely related in the ML tree. The pairwise analysis also shows that the MP similarities cluster mainly according to the virus genus. All of the MPs in this study have been ascribed to the 30K superfamily of viral MPs which share a common aspartic acid residue that is commonly known as the D motif [47,48]. We identified a larger common motif in the emaraviruses and tospoviruses Φ-X-Φ-P-X_(15)_-D-X_(53–59)_-W, while the tenuiviruses have a submotif Φ-X-Φ-P-D. 

Until now horizontal gene transfer among positive-strand RNA viruses and double-strand RNA viruses has been well described but there has been little evidence of gene exchanges among negative-strand RNA viruses [14,60,61]. Horizontal gene exchanges among families within *Bunyavirales* might occur by recombination or reassortment of segments [55,57,62]. Many viruses that infect plants or vertebrates have an insect vector that is responsible for transmission, or that can also serve as an alternative host supporting virus replication. We conducted co-phylogeny analysis to investigate the possibility of segment reassortment or recombination occurring between virus species in common ancestor hosts. Across all families, duplication and host switching were more common than co-divergence with a host species. Looking at the families containing plant viruses, *Fimoviridae* shows duplication, and host switching occurs more than co-divergence while *Tospoviridae* and *Phenuiviridae* show very little host switching. Extinction was high for most families in *Bunyavirales* and this outcome can occur if there is an incongruency between the virus and host phylogenies, when invertebrate vectors narrow the niche diversity, or spill-over infection leads to a dead-end [58,59,63,64]. Extinction may also appear high if the virus and/or host have recently emerged. To better understand the co-phylogeny, we overlaid the plant hosts and insect vector on an ML tree of the plant infecting tenuiviruses, orthotospoviruses, and emaraviruses [65]. The tenuiviruses have four to six genome segments, infect only monocots, and are vectored by hemipteran insects (plant hoppers). The presence of a large segmented genome and its recent origin might have reduced the opportunities for a broader invertebrate vector range. The tospoviruses and emaraviruses infect Superrosids and Superastrids and have clearly separate insect and arachnid vectors. The opportunities for heterotypic reassortment between these genera would more likely occur in a common host than a common vector. Considering the evolutionary history of superrosids and superasterids, these represent two large clades of eudicot plants that emerged approximately 5 million years ago [33,49]. Orthotospoviruses and emaraviruses include a number of recently emerged virus species [43,66,67]. Their emergence may be due to recent commercial trade enabling viruses to move into new geographic regions without expanding the host species diversity. Importantly, the lack of evidence for strong co-speciation argues for a shallow evolutionary clock which may make this study a poor fit for the data.

Our findings provide a comprehensive view of plant virus phylogenetic relationships within the higher ranking of the order *Bunyavirales*. The phylogenies reveal extensive conservation among the hallmark genes of plant-infecting viruses with insect and vertebrate counterparts. The phylogenies reveal important insights into the strength of virus–host and virus–vector interactions. Further research is needed to understand the potential for horizontal gene transfer across diverse virus lineages. A priority for future research is to understand the barriers to virus and host co-speciation that could be critical for preventing epidemic virus spread.

## Supplementary Materials

The following are available online at https://www.mdpi.com/1999-4915/12/9/1010/s1, Figure S1: Extracted interior branches of a monophyletic group representing *Chilibre phlebovirus* and neighboring *Pacuvirus* species. Branch support values obtained using the aLRT-SH-like test are provided. All panels show 100% support for monophyletic grouping between *Pacuvirus* and *Chilibre phlebovirus*. A. is the RdRp phylogeny in Figure 1, B. is the preGP phylogeny in Figure 3 and, C. is the N protein phylogeny in Figure 4. Figure S2: Genome structure of M segments from type species of each genera belonging to the order Bunyavirales; Figure S3: Multiple sequence alignment of domains from movement protein (MP) of plant viruses within the order *Bunyavirales*. Table S1: Genome structure, nucleotide, and protein accession numbers of the segments and viral proteins used in this study; Table S2: The 20 distant nucleotides of 5′ and 3′ termini belonging to L, M, and S segments of *Bunyavirales*; Table S3: The 5 distant nucleotides of 5′ and 3′ termini belonging to L, M, and S segments of *Bunyavirales* that used to generate consensus sequences; Table S4: Host species information of the *Bunyavirales* viruses.

## References

[1] Wichgers Schreur P.J., Kormelink R., Kortekaas J. (2018). Genome packaging of the Bunyavirales. Curr. Opin. Virol..

[2] Abudurexiti A., Adkins S., Alioto D., Alkhovsky S.V., Avšič-Županc T., Ballinger M.J., Bente D.A., Beer M., Bergeron É., Blair C.D. (2019). Taxonomy of the order Bunyavirales: Update 2019. Arch. Virol..

[3] Navarro B., Minutolo M., De Stradis A., Palmisano F., Alioto D., Di Serio F. (2018). The first phlebo-like virus infecting plants: A case study on the adaptation of negative-stranded RNA viruses to new hosts. Mol. Plant Pathol..

[4] Navarro B., Zicca S., Minutolo M., Saponari M., Alioto D., Di Serio F. (2018). A negative-stranded RNA virus infecting citrus trees: The second member of a new genus within the order bunyavirales. Front. Microbiol..

[5] Li C.X., Shi M., Tian J.H., Lin X.D., Kang Y.J., Chen L.J., Qin X.C., Xu J., Holmes E.C., Zhang Y.Z. (2015). Unprecedented genomic diversity of RNA viruses in arthropods reveals the ancestry of negative-sense RNA viruses. Elife.

[6] Guterres A., de Oliveira R.C., Fernandes J., de Lemos E.R.S., Schrago C.G. (2017). New bunya-like viruses: Highlighting their relations. Infect. Genet. Evol..

[7] Ballinger M.J., Taylor D.J. (2019). Evolutionary persistence of insect bunyavirus infection despite host acquisition and expression of the viral nucleoprotein gene. Virus Evol..

[8] Whitfield A.E., Huot O.B., Martin K.M., Kondo H., Dietzgen R.G. (2018). Plant rhabdoviruses—Their origins and vector interactions. Curr. Opin. Virol..

[9] Bojko J., Subramaniam K., Waltzek T.B., Stentiford G.D., Behringer D.C. (2019). Genomic and developmental characterisation of a novel bunyavirus infecting the crustacean *Carcinus maenas*. Sci. Rep..

[10] Käfer S., Paraskevopoulou S., Zirkel F., Wieseke N., Donath A., Petersen M., Jones T.C., Liu S., Zhou X., Middendorf M. (2019). Re-assessing the diversity of negative strand RNA viruses in insects. PLoS Pathog..

[11] Koonin E.V., Dolja V.V. (2014). Virus World as an Evolutionary Network of Viruses and Capsidless Selfish Elements. Microbiol. Mol. Biol. Rev..

[12] Kormelink R., Garcia M.L., Goodin M., Sasaya T., Haenni A.L. (2011). Negative-strand RNA viruses: The plant-infecting counterparts. Virus Res..

[13] Simmonds P., Adams M.J., Benk M., Breitbart M., Brister J.R., Carstens E.B., Davison A.J., Delwart E., Gorbalenya A.E., Harrach B. (2017). Consensus statement: Virus taxonomy in the age of metagenomics. Nat. Rev. Microbiol..

[14] Wolf Y.I., Kazlauskas D., Iranzo J., Lucía-Sanz A., Kuhn J.H., Krupovic M., Dolja V.V., Koonin E.V. (2018). Origins and evolution of the global RNA virome. mBio.

[15] Kuraku S., Zmasek C.M., Nishimura O., Katoh K. (2013). aLeaves facilitates on-demand exploration of metazoan gene family trees on MAFFT sequence alignment server with enhanced interactivity. Nucleic Acids Res..

[16] Katoh K., Rozewicki J., Yamada K.D. (2019). MAFFT online service: Multiple sequence alignment, interactive sequence choice and visualization. Brief. Bioinform..

[17] MAFFT Version 7. https://mafft.cbrc.jp/alignment/server/.

[18] Capella-Gutierrez S., Silla-Martinez J.M., Gabaldon T. (2009). trimAl: A tool for automated alignment trimming in large-scale phylogenetic analyses. Bioinformatics.

[19] Chen C., Chen H., Zhang Y., Thomas H.R., Frank M.H., He Y., Xia R. (2020). TBtools—An integrative toolkit developed for interactive analyses of big biological data. Mol. Plant.

[20] Darriba D., Taboada G.L., Doallo R., Posada D. (2011). ProtTest 3: Fast selection of best-fit models of protein evolution. Bioinformatics.

[21] Guindon S., Delsuc F., Dufayard J.-F., Gascuel O., Posada D. (2009). Estimating Maximum Likelihood Phylogenies with PhyML. Bioinformatics for DNA Sequence Analysis.

[22] Gouy M., Guindon S., Gascuel O. (2010). SeaView Version 4: A multiplatform graphical user Interface for sequence alignment and phylogenetic tree building. Mol. Biol. Evol..

[23] Letunic I., Bork P. (2019). Interactive Tree Of Life (iTOL) v4: Recent updates and new developments. Nucleic Acids Res..

[24] iTOL. https://itol.embl.de/.

[25] Lorenz R., Bernhart S.H., Höner zu Siederdissen C., Tafer H., Flamm C., Stadler P.F., Hofacker I.L. (2011). ViennaRNA Package 2.0. Algorithms Mol. Biol..

[26] Crooks G.E., Hon G., Chandonia J.-M., Brenner S.E. (2004). WebLogo: A sequence logo generator. Genome Res..

[27] WebLogo 3. http://weblogo.threeplusone.com/.

[28] Muhire B.M., Varsani A., Martin D.P. (2014). SDT: A virus classification tool based on pairwise sequence alignment and identity calculation. PLoS ONE.

[29] Conow C., Fielder D., Ovadia Y., Libeskind-Hadas R. (2010). Jane: A new tool for the cophylogeny reconstruction problem. Algorithms Mol. Biol..

[30] Taxonomy. https://www.ncbi.nlm.nih.gov/taxonomy.

[31] Virus-Host Database. https://www.genome.jp/virushostdb/.

[32] Mihara T., Nishimura Y., Shimizu Y., Nishiyama H., Yoshikawa G., Uehara H., Hingamp P., Goto S., Ogata H. (2016). Linking virus genomes with host taxonomy. Viruses.

[33] Chase M.W., Christenhusz M.J.M., Fay M.F., Byng J.W., Judd W.S., Soltis D.E., Mabberley D.J., Sennikov A.N., Soltis P.S., Stevens P.F. (2016). An update of the Angiosperm Phylogeny Group classification for the orders and families of flowering plants: APG IV. Bot. J. Linn. Soc..

[34] ANGIOSPERM PHYLOGENY WEBSITE, Version 14. http://www.mobot.org/MOBOT/research/APweb/.

[35] Mielke-Ehret N., Mühlbach H.-P. (2012). Emaravirus: A novel genus of multipartite, negative strand RNA plant viruses. Viruses.

[36] German T.L., Lorenzen M.D., Grubbs N., Whitfield A.E. (2020). New technologies for studying negative-strand RNA viruses in plant and arthropod hosts. Mol. Plant Microbe Interact..

[37] Dolja V.V., Koonin E.V. (2011). Common origins and host-dependent diversity of plant and animal viromes. Curr. Opin. Virol..

[38] Amroun A., Priet S., de Lamballerie X., Quérat G. (2017). Bunyaviridae RdRps: Structure, motifs, and RNA synthesis machinery. Crit. Rev. Microbiol..

[39] Ferron F., Weber F., de la Torre J.C., Reguera J. (2017). Transcription and replication mechanisms of Bunyaviridae and Arenaviridae L proteins. Virus Res..

[40] Sun Y., Li J., Gao G.F., Tien P., Liu W. (2018). Bunyavirales ribonucleoproteins: The viral replication and transcription machinery. Crit. Rev. Microbiol..

[41] Guardado-Calvo P., Rey F.A. (2017). The Envelope Proteins of the Bunyavirales. Advances in Virus Research.

[42] Brown J.K., Fauquet C.M., Briddon R.W., Zerbini M., Moriones E., Navas-Castillo J. (2012). Bunyaviridae. Virus Taxonomy.

[43] Ishikawa K., Maejima K., Komatsu K., Netsu O., Keima T., Shiraishi T., Okano Y., Hashimoto M., Yamaji Y., Namba S. (2013). Fig mosaic emaravirus p4 protein is involved in cell-to-cell movement. J. Gen. Virol..

[44] Yu C., Karlin D.G., Lu Y., Wright K., Chen J., MacFarlane S. (2013). Experimental and bioinformatic evidence that raspberry leaf blotch emaravirus P4 is a movement protein of the 30K superfamily. J. Gen. Virol..

[45] Xin M., Cao M., Liu W., Ren Y., Zhou X., Wang X. (2017). Two negative-strand RNA viruses identified in watermelon represent a novel clade in the order Bunyavirales. Front. Microbiol..

[46] Zhao W., Jiang L., Feng Z., Chen X., Huang Y., Xue F., Huang C., Liu Y., Li F., Liu Y. (2016). Plasmodesmata targeting and intercellular trafficking of Tomato spotted wilt tospovirus movement protein NSm is independent of its function in HR induction. J. Gen. Virol..

[47] Melcher U. (2000). The “30K” superfamily of viral movement proteins. J. Gen. Virol..

[48] Mushegian A.R., Elena S.F. (2015). Evolution of plant virus movement proteins from the 30K superfamily and of their homologs integrated in plant genomes. Virology.

[49] Moore M.J., Soltis P.S., Bell C.D., Burleigh J.G., Soltis D.E. (2010). Phylogenetic analysis of 83 plastid genes further resolves the early diversification of eudicots. Proc. Natl. Acad. Sci. USA.

[50] Terret-Welter Z., Bonnet G., Moury B., Gallois J.-L. (2020). Analysis of tomato spotted wilt virus RNA-dependent RNA polymerase adaptative evolution and constrained domains using homology protein structure modelling. J. Gen. Virol..

[51] Klemm C., Reguera J., Cusack S., Zielecki F., Kochs G., Weber F. (2013). Systems to establish Bunyavirus genome replication in the absence of transcription. J. Virol..

[52] Guo Y., Liu B., Ding Z., Li G., Liu M., Zhu D., Sun Y., Dong S., Lou Z. (2017). Distinct mechanism for the formation of the ribonucleoprotein complex of Tomato spotted wilt virus. J. Virol..

[53] Suzuki Y., Kobayashi Y. (2013). Evolution of complementary nucleotides in 5’ and 3’ untranslated regions of influenza A virus genomic segments. Infect. Genet. Evol..

[54] Hughes H.R., Russell B.J., Lambert A.J. (2020). Genetic characterization of frijoles and chilibre species complex viruses (genus phlebovirus; Family phenuiviridae) and three unclassified new world phleboviruses. Am. J. Trop. Med. Hyg..

[55] Briese T., Calisher C.H., Higgs S. (2013). Viruses of the family Bunyaviridae: Are all available isolates reassortants?. Virology.

[56] Wang J., Firth C., Amos-Ritchie R., Davis S.S., Yin H., Holmes E.C., Blasdell K.R., Walker P.J. (2019). Evolutionary history of Simbu serogroup orthobunyaviruses in the Australian episystem. Virology.

[57] Roossinck M.J., Ali A. (2007). Mechanisms of plant virus evolution and identification of genetic bottlenecks: Impact on disease management. Biotechnology and Plant Disease Management.

[58] Shi M., Lin X.D., Tian J.H., Chen L.J., Chen X., Li C.X., Qin X.C., Li J., Cao J.P., Eden J.S. (2016). Redefining the invertebrate RNA virosphere. Nature.

[59] Holmes E.C., Zhang Y.Z. (2015). The evolution and emergence of hantaviruses. Curr. Opin. Virol..

[60] Koonin E.V., Dolja V.V. (2012). Expanding networks of RNA virus evolution. BMC Biol..

[61] Liu H., Fu Y., Jiang D., Li G., Xie J., Cheng J., Peng Y., Ghabrial S.A., Yi X. (2010). Widespread Horizontal Gene Transfer from Double-Stranded RNA Viruses to Eukaryotic Nuclear Genomes. J. Virol..

[62] Webster C.G., Reitz S.R., Perry K.L., Adkins S. (2011). A natural M RNA reassortant arising from two species of plant- and insect-infecting bunyaviruses and comparison of its sequence and biological properties to parental species. Virology.

[63] Geoghegan J.L., Senior A.M., Di Giallonardo F., Holmes E.C. (2016). Virological factors that increase the transmissibility of emerging human viruses. Proc. Natl. Acad. Sci. USA.

[64] Geoghegan J.L., Duchêne S., Holmes E.C. (2017). Comparative analysis estimates the relative frequencies of co-divergence and cross-species transmission within viral families. PLoS Pathog..

[65] Domingo E. (2000). Viruses at the Edge of Adaptation. Virology.

[66] McGavin W.J., Mitchell C., Cock P.J.A., Wright K.M., MacFarlane S.A. (2012). Raspberry leaf blotch virus, a putative new member of the genus Emaravirus, encodes a novel genomic RNA. J. Gen. Virol..

[67] Yang C., Zhang S., Han T., Fu J., Di Serio F., Cao M. (2019). Identification and characterization of a novel emaravirus associated with jujube (*Ziziphus jujuba* Mill.) yellow mottle disease. Front. Microbiol..

