# Supplementary figures and images for: Family Level Phylogenies Reveal Relationships of Plant Viruses within the Order Bunyavirales
Source: Viruses. 2020 Sep 10;12(9):1010. doi: 10.3390/v12091010 (PMC7551631; doi:10.3390/v12091010)

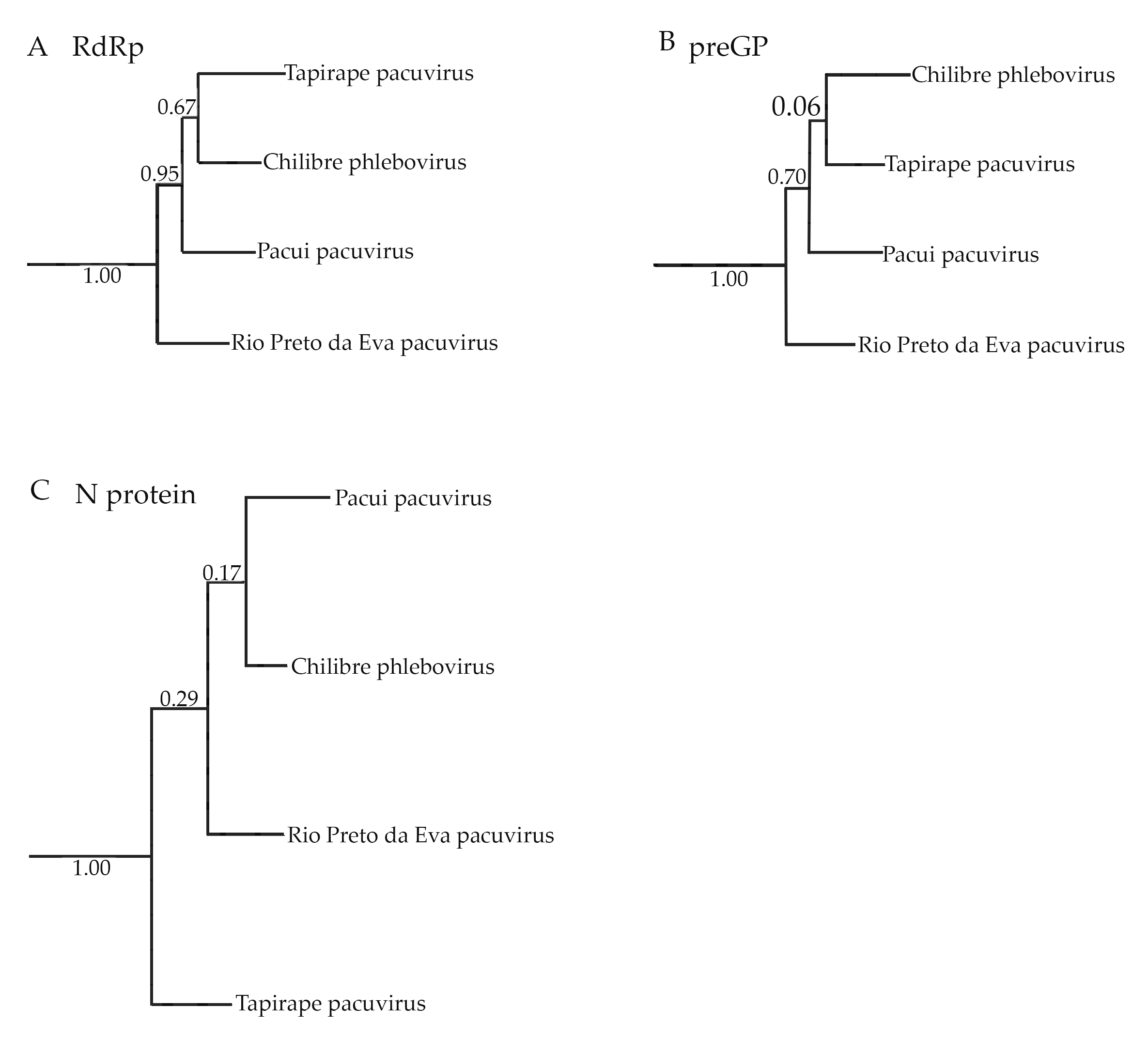

Supplement: Supplementary file 1 [file viruses-12-01010-s001.zip › Supplementary Fig. S1 Chilibrae.tif]

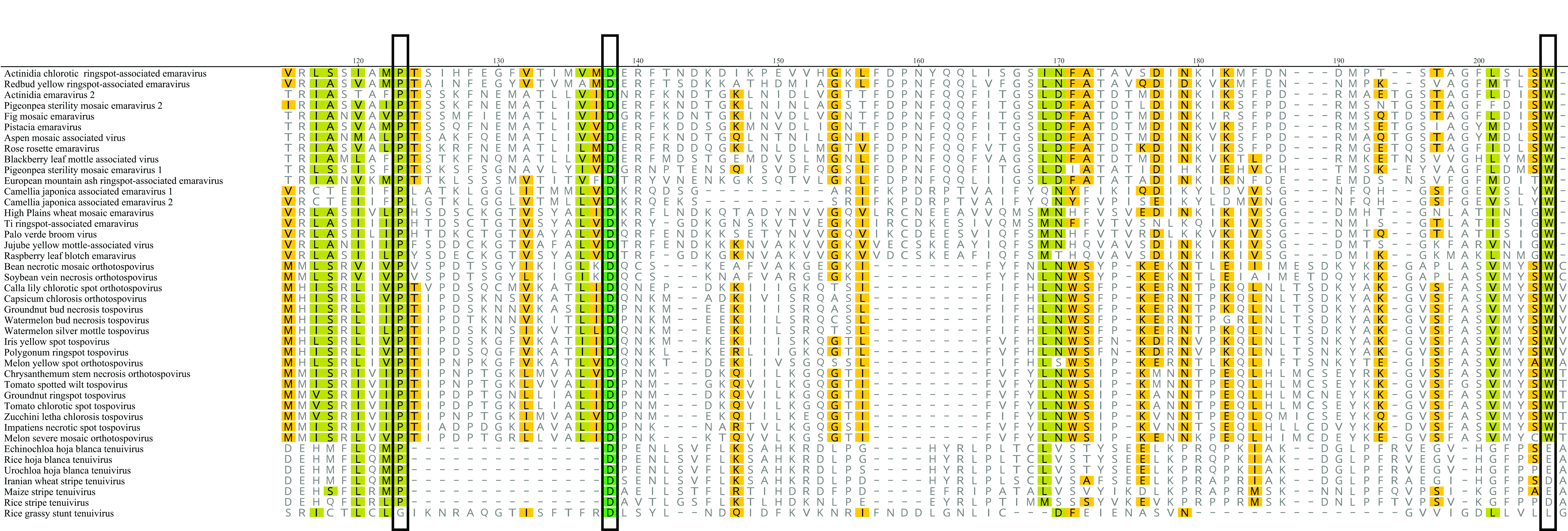

Supplement: Supplementary file 1 [file viruses-12-01010-s001.zip › Supplementary Fig. S3 MP Domain.tif]
